# Experienced and Anticipated Intersectional Violence and Psychological Distress Symptom Severity Among Black Transgender Women in the United States of America

**DOI:** 10.3390/healthcare14070932

**Published:** 2026-04-02

**Authors:** Athena D. F. Sherman, Monique S. Balthazar, Ashley M. Ruiz, Diane Berish, Molly Szczech, Sarah Wishloff, Jordan Pelkmans, GaEun Kim, Jason S. Schneider, Don Operario, Together We Thrive Community Advisory Board, Andrea N. Cimino

**Affiliations:** 1 Nell Hodgson Woodruff School of Nursing, Emory University, Atlanta, GA 30322, USA; 2 Ross and Carol Nese College of Nursing, The Pennsylvania State University, University Park, PA 16802, USA; msb6243@psu.edu (M.S.B.);; 3 School of Medicine, Emory University, Atlanta, GA 30322, USA; 4 College of Health Solutions, Arizona State University, Phoenix, AZ 85004, USA; gakim1@asu.edu; 5 Rollins School of Public Health, Emory University, Atlanta, GA 30322, USA; 6 Together We Thrive: Research and Education Group, Atlanta, GA 30084, USA; 7 Rogue Scholar Consulting, Baltimore, MD 21202, USA

**Keywords:** PTSD, psychological distress, depression, transgender, racism, violence, sexual violence, anticipated violence, internalized stigma, cisgenderism, intersectionality

## Abstract

**Highlights:**

**What are the main findings?**

- Experienced intersectional sexual violence and anticipation of violence remained significantly associated with PTSD and depressive symptom severity in separate models when controlling for age, employment, United States region, and other forms of intersectional violence.

**What are the implications of the main findings?**

- The specificity of sexual violence’s association with symptom severity may reflect the particularly traumatic and identity-threatening nature of this form of victimization among Black transgender women and the internalization of future risks of sexual assault.
- Moreover, findings suggest that the expectation of future physical victimization based on one’s intersectional identities constitutes a stressor with measurable associations with mental health symptom severity—reflecting probable internalized processes of vigilance and fear that emerge from repeated exposure to stigma and discrimination.

**Abstract:**

Background: Black transgender women experience disproportionately high rates of violent victimization rooted in intersecting systems of oppression, including cisgenderism and anti-Black racism. Although victimization is linked to psychological distress, the mental health impacts of intersectional violence, which targets overlapping marginalized identities, remain understudied. Objectives: To examine the associations between anticipated and experienced intersectional victimization and psychological distress among Black transgender women. Methods: Online survey data from 151 Black transgender women (age ≥ 18) in the United States (US) between October 2021 and February 2024 were analyzed using *t*-tests and multivariate linear regressions. Results: In models controlling for age, employment, and US region, experienced sexual, physical, and threats of intersectional violence, as well as anticipated intersectional violence, were associated with increased post-traumatic stress disorder (PTSD) symptom severity, in separate models. Conversely, only experienced sexual intersectional violence and anticipated intersectional violence were associated with greater depressive symptom severity. When all violence variables were included simultaneously, experienced intersectional sexual violence and anticipated violence remained significantly associated with PTSD and depressive symptoms in separate models. Conclusions: Service providers who work with Black transgender women should routinely assess for anticipated and experienced intersectional victimization to guide person-centered interventions. Further research is needed to distinguish the effects of intersectional victimization from opportunistic victimization and to inform the adaptation of targeted mental health interventions.

## 1. Introduction

Transgender women, defined as women or gender-diverse individuals identifying as transfeminine and assigned male sex at birth, experience high rates of discrimination and violent victimization, often targeting their gender minority status and subversion of societal gender norms [1,2,3,4]. The 2015 United States Transgender Survey (USTS) found that 48% of transgender and gender-diverse (TGD) participants (*n* = 27,715) reported being denied equal treatment or service, verbally harassed, and/or physically attacked because of their gender identity in the previous year [5]. Victimization often begins early in life and reoccurs throughout the lifespan, with childhood gender-based violence affecting an estimated 27–69% of transgender individuals in the United States (US) [6]. Relative to other sexual and gender minorities (SGMs), transgender women are at an increased risk of repeated and multidimensional violence, including interpersonal (physical and sexual violence), material (economic and legal discrimination), and structural or symbolic forms (domination and control exerted through sociocultural norms, practices, and policies) [7,8,9,10].

Given their multiple marginalized positionalities, Black transgender women face a heightened risk of polyviolence (multiple, intersecting forms of violence) and associated mental health disparities compared to White transgender women [1,11,12,13,14,15]. In the 2015 USTS, Black transgender respondents were more likely than their White counterparts to report sexual assault and unequal treatment in the past year [5]. Prior research shows that victimization is strongly associated with psychological distress among transgender women, including symptoms of depression [16,17,18], PTSD [12], suicidality [19,20,21], anxiety [22,23], and chronic stress [24]. In the USTS, 41% of Black transgender respondents reported serious psychological distress in the prior month compared to 5% of the overall US population [15]. Anticipated stigma (fear of discrimination or vigilance in anticipation of future victimization) is also linked to deleterious mental health effects and allostatic load [25,26]. These findings align with broader evidence that discrimination and chronic stigma contribute to mental health disparities among people with marginalized identities [27,28,29].

To date, much of the research examining the association between victimization and psychological distress in TGD populations has focused on overt forms of violence, such as physical violence and/or sexual assault [6]. The literature on intersectional or anticipated gender-based victimization against TGD people is comparatively limited [30]. Although TGD people are exposed to violence and experience severe mental health symptoms at higher rates than both the overall population and other SGM, there are even fewer studies exploring victimization as a predictor of mental health outcomes among Black transgender women [14].

Intersectionality, first articulated by Crenshaw (1989) and rooted in Black feminist scholarship, describes how overlapping systems of marginalization (or privilege) based on social categories such as race, ethnicity, sexual orientation, and gender identity, shape lived experiences [31]. Black transgender women occupy multiple axes of oppression, including cisgenderism (the ideology that delegitimizes, pathologizes, and polices gender identities that differ from a person’s assigned sex at birth) [32], transphobia, misogyny, and anti-Black racism. These mutually constitutive forms of social subjugation produce and sustain systemic health inequities [13,33]. For example, data from the National Lesbian Health Care Survey found that violence motivated by victim identity had stronger effects on mental health than intimate partner violence or sexual assault [34]. Other research shows that the combination of transphobia and racism results in higher rates of depression symptoms [35]. Intersectional violence extends beyond individual victims, influencing the broader communities they belong to. In a study of teenagers of color in the US, witnessing violence against people in their community via online videos caused PTSD and depressive symptoms to increase significantly [36]. Thus, while not every TGD individual will experience intersectional violence directly, the impact of each event can affect the entire community.

To eliminate health disparities and develop person-centered, anti-racist mental health interventions, research must more fully incorporate intersectionality in study design and analyses [37]. In response to these gaps and the persistent underrepresentation of Black transgender women in the scientific literature, this study examines associations between anticipated and experienced intersectional victimization and psychological distress among Black transgender women.

## 2. Materials and Methods

A convenience sample of 154 Black transgender women completed an online survey between October 2021 and February 2024. Participants were recruited through in-person and online strategies with guidance from a Community Advisory Board (CAB) of Black transgender women who also contributed to study design, materials, safety protocols, recruitment, member checking of findings, and manuscript development. The study was approved by the Emory University Institutional Review Board (IRB# STUDY00002141). Additional methodological information can be found at Grant and colleagues 2025 [38].

Eligibility criteria included (1) current identification with a feminine gender (woman, transgender woman, transfeminine); (2) assigned male or intersex at birth (and identifying as transgender if intersex); (3) identifying as Black, African American, or African decent—or multi-racial to include one of the previous; (4) residing in the US; (5) aged ≥18 years; and (6) able to read English. To reduce vulnerability to ‘Bots and Nots’, each potential participant was asked to provide a phone number, which was run through an online phone carrier vetting platform used to confirm that the number was a US-based telephone number that was not a voice-over-internet provided number (e.g., Google Voice). Once that criteria was met, a research staff member interviewed all interested parties for eligibility via phone or Zoom. Participants completed a 45 min electronic survey and received a $30 gift card for participation [39,40].

### 2.1. Measures

#### 2.1.1. Demographics

Demographic variables included age, location, income, race, ethnicity, sexual orientation (Straight/Heterosexual, Lesbian, Gay, Bisexual, Queer, Pansexual, Another [fill in the blank]), sex assigned at birth, current gender identity (man, woman, gender diverse, another), health insurance status, education, and employment, among others. Current state and ZIP code were transformed into regions based on the Census Bureau Regions and Divisions [41].

#### 2.1.2. Intersectional Violence

The Intersectional Discrimination Index is a multidimensional self-report tool used to capture anticipated, daily experiences, and major experiences of intersectional discrimination across multiple social identities—with high/moderate internal consistency (*a* = 0.90) test–retest reliability (*r* = 0.70–0.72), and sample invariance among different populations [30,42]. The measure gives the following guidance to participants: “These questions are about experiences related to who you are. This includes both how you describe yourself and how others might describe you. For example, your skin colour, ancestry, nationality, religion, gender, sexuality, age, weight, disability or mental health issue, and income.” Additionally, items begin with the following: “Because of who I am…”. The design of the measure enables participant-reported experiences to be inherently intersectional. Thus, aligning with the state of intersectional measurement, we chose 4 items to examine intersectionality as a lived, holistic experience, acknowledging that co-occurring identities and social positions are non-separable and non-additive.

The 4 items used to examine anticipated intersectional victimization and experienced threats of violence, experienced sexual assault, and experienced physical assault: “… people might try to attack me physically [range: 0 (strongly disagree) to 4 (strongly agree)]; …have you ever been threatened with a physical or sexual attack? [range: 0 (never) to 2 (more than once)]; …have you ever been physically attacked (e.g., spit on, had objects thrown at you, hit, punched, pushed or grabbed, beaten)? [range: 0 (never) to 2 (more than once)]; …have you ever been made to engage in sexual activity, or been touched in a sexual way, that you didn’t want? [range: 0 (never) to 2 (more than once)].” The individual items were operationalized as dichotomous (never vs. once/more than once; strongly disagree/disagree/neutral vs. agree/strongly agree) in *t*-tests and descriptive statistics; and continuous severity ratings for Pearson’s correlations and multiple regression modeling. No cumulative scale or composite variable was constructed to allow for the examination of distinct forms of violence exposure, avoid masking heterogeneity across violence types, and preserve conceptual distinctness of exposures.

#### 2.1.3. PTSD Symptoms

The Post-Traumatic Stress Symptom Checklist (PCL-5) measures the severity of PTSD symptoms within the past month, via a 20-item self-report measure using a 5-point Likert scale (higher scores indicating worse severity)—with high/strong internal consistency (*a* = 0.91–0.96), test–retest reliability (*r* = 0.74–0.90), and convergent validity [43,44,45]. A total score of ≥31 was used to indicate probable PTSD, based on the validation study by Blevins et al. (2015) [43].

#### 2.1.4. Depressive Symptoms

The Beck Depression Inventory II (BDI-II) measures the severity of depressive symptoms in the past two weeks, via a 21-item self-report measure, using a four-point Likert scale (higher scores indicating worse severity)—with high/strong internal consistency (Cronbach’s *a* = 0.90–0.95), test–retest reliability (*r* = 0.93), and convergent validity [46,47]. Total scores from 0 to 13 being “minimal”, 14–19 being “mild”, 20–28 being “moderate, and 29–63 as “severe” depressive symptoms [48]. A cutoff of ≥17 was used to denote clinically relevant depressive symptoms, per the meta-analysis by Von Glischinski et al. (2019) [49]. A clinically important change in symptom severity scores was interpreted as >3 points, per Button et al. 2015 [50].

### 2.2. Data Analyses

Descriptive analyses characterized score distribution and missingness (<5% overall; three cases removed using listwise deletion). Correlations assessed associations among demographic, predictor (intersectional violence) and outcome (mental health) variables. Age, employment, and U.S. region were included as covariates based on significant associations (retained at *p* < 0.10 two-sided) and theoretical relevance based on the minority stress model, which details the context, distal and proximal stressors, and their relationship to health outcomes [27,51]. Multiple regression analyses examined each intersectional discrimination indicator (anticipated violence, threatened violence, sexual violence, or physical violence) as a predictor of total PTSD and Depressive symptoms (eight models total). All regression assumptions were evaluated and met. With a sample size of 151 participants, 4–9 predictors using alpha = 0.05, we were sufficiently powered (beta = 0.8) to detect effect sizes of f^2^ ≈ 0.09–0.11, representing small-to-medium effects [52].

## 3. Results

### 3.1. Sample Characteristics

The final sample consisted of 151 Black transgender women with a mean age of 35.9 years (standard deviation [SD] 11.0); 46.5% lived in the southern US, and 49.6% were currently employed. Most participants (80%) reported at least one form of intersectional victimization: 61.6% agreed or strongly agreed that people might try to attack them physically (from here forward referred to as ‘anticipated violence’), 69.5% experienced threats of physical or sexual attacks (from here forward referred to as ‘experienced threats of violence’), 61.6% experienced physical violence, and 69.5% experienced sexual violence. See Table 1. A full description of the sample and mental health symptoms has been previously published (REDACTED).

### 3.2. Bivariate Analyses

#### 3.2.1. Symptoms of PTSD

Significant positive correlations were found between PTSD and intersectional violence (r = 0.39, *p* < 0.001) and experiences of threats (r = 0.31, *p* < 0.001), physical violence (r = 0.26, *p* < 0.001), and sexual violence (r = 0.43, *p* < 0.001) (see Table 2). Independent *t*-tests revealed statistically and clinically (>10-point mean difference) [43] significant increases in PTSD symptom severity among participants who experienced intersectional (a) threats of violence; (b) physical violence; (c) sexual violence; and (d) those who anticipated intersectional victimization compared to those who did not (see Table 3).

#### 3.2.2. Symptoms of Depression

Significant positive correlations were found between depressive symptom severity and anticipation of intersectional violence (r = 0.34, *p* < 0.001), experiences of threats (r = 0.17, *p* < 0.05), physical violence (r = 0.18, *p* < 0.05), and sexual violence (r = 0.35, *p* < 0.001) (see Table 2). Independent *t*-tests revealed statistically significantly higher depressive symptom severity among participants who experienced intersectional physical violence; and statistically and clinically significant increases (>3-point change) in depressive symptom severity among those who reported (a) sexual violence and (b) those who anticipated intersectional victimization compared to those who did not (see Table 4).

### 3.3. Multiple Regression Analysis PTSD Symptoms

Controlling for age, employment, and US region, experienced and anticipated intersectional victimization was independently associated with PTSD symptom severity (see Table 4). When all four types of violence were entered into a single model, only sexual violence and anticipated intersectional violence remained significant predictors. Experiencing intersectional sexual violence was associated with a 15.7-point increase in PTSD symptom severity (*SE* = 4.0, *p* < 0.001) and anticipating intersectional violence was associated with a 9.0-point increase (*SE* = 3.1, *p* = 0.004). The model explained 26.1% of the variance in PTSD symptoms (F = 5.5, *p* < 0.001).

### 3.4. Multiple Regression Analysis Depression Symptoms

Controlling for age, employment, and US region, only experienced intersectional sexual violence and anticipated intersectional victimization were significantly associated with depressive symptom severity in separate models (see Table 5). When all four types of violence were included together, only sexual violence and anticipated intersectional violence remained significant predictors. Experiencing intersectional sexual violence was associated with a clinically significant increase [43] of a 10.8-point increase in depressive symptom severity (*SE* = 2.5, *p* < 0.001), and anticipating intersectional violence was associated with a 5.1-point increase in depressive symptom severity (*SE* = 2.0, *p* = 0.01). The model overall accounted for 21.2% of the variance in depressive symptoms (F = 4.2, *p* < 0.001).

## 4. Discussion

### 4.1. Intersectional Violence as a Critical Public Health Concern

Research has consistently documented that transgender people of color, particularly Black transgender women, are at higher risk for hate crimes compared to their White counterparts due to these intersectional effects of racism and transphobia [53]. In our convenience sample, nearly all participants (82.7%) experienced at least one form of intersectional victimization and over one-third anticipated future violence, underscoring the pervasive and ongoing threat this population faces, which aligns with prior research documenting extensive victimization among transgender women of color [5,11,53,54]. The intersection of multiple marginalized identities creates compounded vulnerability to violence, with Black transgender women facing heightened risks due to their positioning at the convergence of cisgenderism, transphobia, and anti-Black racism. This phenomenon, termed transmisogynoir, represents a specific form of oppression rooted in anti-Blackness, cissexism, and misogyny that affects Black transgender women [55].

### 4.2. Differential Effects of Intersectional Violence on Mental Health Outcomes

Research examining polyvictimization among transgender women has demonstrated that experiences of multiple types of violence are associated with increased mental health symptoms [11]. Our findings indicate that different forms of intersectional violence have distinct effects on psychological distress. Although all forms of experienced intersectional violence (sexual, physical, and threats) and anticipated violence were associated with increased PTSD symptom severity and several with depressive symptom severity, only experienced sexual intersectional violence and anticipatory violence remained significant predictors of both outcomes when examined simultaneously. This finding is consistent with minority stress theory, which posits that discrimination and victimization based on stigmatized identities contribute to elevated psychological distress among marginalized populations [27,56]. The strong impact of sexual violence on both PTSD and depression may reflect its particularly traumatic and identity-threatening nature, consistent with broader trauma research demonstrating the severe and complex psychological sequelae of sexual assault [57].

### 4.3. The Role of Anticipated Violence in Psychological Distress

A notable finding is the significant association between anticipated intersectional violence and increased PTSD and depressive symptom severity, indicating that the expectation of future victimization is itself a psychological stressor. In minority stress theory, anticipated discrimination represents a proximal stressor, reflecting internalized vigilance and fear that emerge from repeated exposure to stigma and discrimination [27,56]. Prior research links expectations of rejection and anticipated stigma with poor mental health among transgender populations [26,58], and our results extend this to show its specific impact on Black transgender women’s PTSD symptoms. The psychological impact of anticipated violence may be particularly pronounced among Black transgender women due to the documented high rates of fatal violence against this population [53], which may contribute to heightened vigilance and expectations of future victimization, including fatal violence. Therefore, interventions addressing mental health among Black transgender women should consider not only past and current experiences of violence but also future-oriented fears and expectations of victimization.

### 4.4. Clinical Implications for Mental Health Assessment and Intervention

Given that 52% of participants met criteria for probable PTSD and 45% met criteria for moderate-to-extreme depression, the mental health burden among Black transgender women is substantial and warrants specialized clinical attention. The differential associations between types of intersectional violence and mental health outcomes suggest that trauma-informed interventions may need to be tailored based on specific violence experiences, with particular attention to sexual violence and anticipated victimization.

For Black transgender women experiencing trauma from intersectional sexual violence, evidence-based approaches such as trauma-focused Cognitive–Behavioral Therapy [59] and adapted Cognitive Processing Therapy [60] may help address maladaptive beliefs about danger and self-blame while affirming gender identity. Clinicians should validate that hypervigilance and anticipatory fear are rational responses to documented rates of violence against Black transgender women [54], rather than pathologizing adaptive caution. Interventions should distinguish between protective vigilance and debilitating hyperarousal through safety planning, psychoeducation about trauma responses, and community-based resilience strategies that foster agency and meaningful engagement [61].

### 4.5. Structural, Systemic, and Epistemic Drivers of Intersectional Victimization

Intersectional frameworks illuminate how interconnected social positionalities of vulnerability [62,63] and the epistemic assumptions that shape whose experiences are believed or dismissed, interact with racism, cisgenderism, misogyny, and transphobia to create unique vulnerabilities throughout the lifespan. The high prevalence of violence found in this study underscores the need for structural, systemic and epistemic interventions to address the root causes of violence against Black transgender women. Broader systems of oppression, including employment discrimination, housing instability, and healthcare barriers, intensify vulnerability among transgender women of color [64] and continue to shape present-day social conditions [65,66,67,68]. These dynamics are especially pronounced in the sociopolitical context of the US South, where long-standing racial and gender ideologies have historically denied Black women protection from interpersonal violence.

Secondary victimization can occur when structural and systemic conditions within formal institutions recreate additional harm for individuals with previous victimization histories [69,70,71]. For example, poor treatment that wrongly casts doubt or a lack of credibility towards a person’s knowledge, refusal to recognize a ‘victim’ as knowing their interests or creating distortions of societal understandings of the victim’s realities [72]. For Black transgender women, the anticipation and experience of intersectional victimization in formal healthcare delivery jeopardizes health opportunities, can worsen pre-existing health disparities, and deter individuals from seeking available health resources [69,73].

Reducing the mental health impacts of intersectional violence requires structural and policy interventions addressing root causes. The pervasive anticipatory fear documented in this study—with 62% of participants expecting future victimization—reflects structural inequity and insufficient legal protections for Black transgender women. Comprehensive nondiscrimination protections in hate crime statutes, e employment, housing, and healthcare, combined with law enforcement reforms and accountability mechanisms, are essential to reducing both actual violence and chronic anticipatory stress [74]. Trauma-informed training for healthcare providers and first responders that addresses transmisogynoir and systemic racism can reduce secondary victimization and foster the safety necessary for Black transgender women to seek care. Additionally, addressing social determinants (employment discrimination, housing instability, poverty, and barriers to identity documentation) reduces survival-driven engagement in criminalized economies and exposure to violence [64]. Ultimately, clinical care alone cannot resolve health disparities rooted in systemic oppression; achieving health equity requires dismantling the intersecting systems of anti-Black racism and cisgenderism that produce both violence and anticipated victimization [75,76].

### 4.6. Limitations and Strengths

Several study limitations should be acknowledged. First, the cross-sectional design precludes conclusions about causality between forms of intersectional violence and mental health outcomes; longitudinal research is needed to clarify temporal sequencing and directional effects. Second, community-based convenience sampling may limit generalizability, especially for Black transgender women who are more socially isolated or less connected to LGBTQ+ community organizations. Third, although the sample size is relatively robust for research with this population, it is nonetheless limited in statistical power to detect smaller effects. Reliance on self-report introduces recall bias, social desirability, and participants’ varying interpretations of sensitive questions. Additionally, funding constraints limited our ability to provide the survey in languages other than English, which reduces the generalizability of our findings.

Additionally, the PCL-5 and BDI-II were normed in samples without Black transgender women (military veterans and psychiatric outpatients, respectively), so their psychometric properties may not be equivalent for this population. Some PTSD symptoms (e.g., hypervigilance) may be adaptive to ongoing threats rather than solely pathological, and depressive symptoms may not fully capture culturally specific distress or responses to systemic oppression. Clinical cutoff scores derived from predominantly White samples may result in misclassification of Black transgender women.

Further, while the Intersectional Discrimination Index items were specifically designed to capture holistic lived experiences of people in a non-additive fashion, the self-report method cannot definitively attribute causation to intersecting identities versus single-axis motivations. Participants may perceive violence as targeting multiple marginalized identities simultaneously, but perpetrators’ actual motivations are often unknown or ambiguous [30]. This reflects epistemological challenges in quantitatively operationalizing intersectionality while maintaining that social categories are mutually constitutive rather than additive [77]. However, victim perception of intersectional targeting may itself be clinically and theoretically meaningful, as the subjective experience of identity-based violence shapes trauma responses regardless of perpetrator intent. Despite these constraints, several strengths enhance the study’s contribution. Centering Black transgender women and using a geographically diverse, community-engaged sample guided by a community advisory board strengthens cultural validity. Analytically, the study identified the unique association of sexual violence with PTSD and depressive symptoms, offering insight into the particularly identity-threatening nature of sexualized harm in this population. The finding that anticipated future violence also predicts symptom severity underscores the psychological toll of chronic vigilance and threat rooted in intersecting stigmas. Collectively, these contributions advance intersectional trauma research by examining both experienced and anticipated violence with validated measures and robust analytic approaches.

### 4.7. Cross-Cultural Validity and Generalizability

While this study makes an important contribution to understanding intersectional violence among Black transgender women, its generalizability is limited to the US context. The sample was recruited from across US regions, and the theoretical frameworks applied—including minority stress theory [27], intersectionality theory [31], and transmisogynoir [55]—are grounded in US sociopolitical histories of anti-Black racism, cisgenderism, and transphobia. Cross-cultural research suggests that experiences of gender-based violence among transgender women vary substantially across national contexts due to differing legal protections, cultural attitudes toward gender diversity, and healthcare systems [78]. For example, transgender women in Latin America face disproportionate rates of fatal violence [79], with different patterns of victimization than those documented in the US. Therefore, the specific manifestations of intersectional violence documented in this US-based study—including the prominence of sexual violence and anticipated victimization among Black transgender women—may not generalize to other nations or ethnic groups. Future research should examine violence against transgender women and gender-expansive people in other countries and cultural contexts to illuminate both universal and context-specific dimensions of victimization and to inform culturally tailored interventions globally. Additionally, future research should examine the latent structure and co-occurrence of the violence exposures examined in this study (e.g., factor analysis, person-centered approaches) and could compare item-level versus composite or interaction-based approaches to improve the measurement and operationalization of intersectional violence.

## 5. Conclusions

This study addresses a critical gap in the literature focused on the relationship between intersectional violence and mental health outcomes, particularly among Black transgender women. Given the disproportionate pervasiveness of violence in the lives of Black transgender women, intersectional inquiry offers contextualization to differing forms of risk and exposure to different types of victimization across interpersonal–structural levels of society [80]. Ultimately, the epistemic injustice of anticipated and experienced forms of intersectional victimization on psychological distress outcomes among Black transgender women calls for needed healthcare research and delivery informed by intersectional inquiry.

## Figures and Tables

**Table 1:** Participant Demographics (*N* = 151).

Characteristic	*n* (%)/*M* (*SD*)
Age Group	
18–24 years	19 (12.6%)
25–34 years	58 (38.4%)
35–64 years	73 (48.3%)
65+ years	1 (0.7%)
Age	35.93 (10.98)
Sexual Identity	
Straight/Heterosexual	54 (35.8%)
Lesbian	9 (6.0%)
Gay	26 (17.2%)
Bisexual	12 (7.9%)
Queer	18 (11.9%)
Pansexual	19 (12.6%)
Another	13 (8.6%)
Race ()	
Black	139 (92.1%)
Multiple races to include Black	12 (7.9%)
Ethnicity	
Hispanic/Latinx	21 (13.9%)
Non-Hispanic/Latinx	130 (86.1%)
Region	
South	70 (46.4%)
Northeast	29 (19.2%)
Midwest	27 (17.9%)
West	24 (15.9%)
Education	
Did not complete high school	18 (11.9%)
High school diploma/GED	68 (45.0%)
Technical/vocational/trade school	13 (8.6%)
Associate degree	13 (8.6%)
Bachelor’s degree	24 (15.9%)
Some graduate school	10 (6.6%)
Master’s degree	4 (2.6%)
PhD	1 (0.7%)
Employment Status	
Unemployed	77 (51.0%)
Employed full-time	49 (32.5%)
Employed part-time	25 (16.6%)
Work Status (detailed)	
Employed full-time	38 (25.2%)
Employed part-time	14 (9.3%)
Self-employed	9 (6.0%)
Street economy	5 (3.3%)
Homemaker	4 (2.6%)
On disability	19 (12.6%)
On public assistance	1 (0.7%)
Student (full-time)	1 (0.7%)
Unemployed <1 year	15 (9.9%)
Unemployed 1+ years	7 (4.6%)
Multiple job categories	38 (25.2%)
Income annually	
$0–$9999	56 (37.1%)
$10,000–$19,999	37 (24.5%)
$20,000–$39,999	31 (20.5%)
$40,000–$59,999	20 (13.2%)
$60,000–$79,999	4 (2.6%)
$80,000+	3 (2.0%)
Health Insurance	
No insurance	15 (9.9%)
Employee health plan	24 (15.9%)
Parents’ insurance	11 (7.3%)
Private purchase	6 (4.0%)
Medicare/Medicaid	85 (56.3%)
Student insurance	2 (1.3%)
Other	8 (5.3%)
Food Insecurity	
Never hungry	60 (39.7%)
Rarely hungry	59 (39.1%)
Hungry many days	32 (21.2%)
Violence Variables (binary)	
Threat of violence (once/more than once)	105 (69.5%)
Physical violence (once/more than once)	93 (61.6%)
Sexual violence (once/more than once)	105 (69.5%)
Anticipated violence (agree/strongly agree)	93 (61.6%)
Mental Health Variables	
PTSD diagnosis (PCL-5 > 31)	78 (51.7%)
Depression category (BDI-II)	
Normal ups/downs	46 (30.5%)
Mild	21 (13.9%)
Borderline	16 (10.6%)
Moderate	39 (25.8%)
Severe	23 (15.2%)
Extreme	5 (3.3%)
Mean symptom scores	
PCL-5 total (SD)	28.85 (19.72)
BDI-II total (SD)	18.70 (12.17)

**Table 2:** Pearson’s Correlation (*N* = 151).

	1	2	3	4	5	6	7
1. Anticipated Violence		0.394 ***	0.366 ***	0.374 ***	0.394 ***	0.338 ***	−0.158
2. Threatened with a physical or sexual attack			0.596 ***	0.634 ***	0.306 ***	0.166 *	−0.162 *
3. Physical Violence				0.484 ***	0.262 ***	0.175 *	0.06
4. Sexual Violence					0.428 ***	0.349 ***	−0.174 *
5. PTSD (*a* = 0.96)						0.773 ***	−0.089
6. Depression (*a* = 0.90)							−0.072
7. Age							

**Note.** *** Correlation is significant at the 0.001 level (2-tailed). * Correlation is significant at the 0.05 level (2-tailed). PTSD = post-traumatic stress disorder symptom severity.

**Table 3:** Independent Sample *t*-Tests.

**Differences in Depressive Symptoms (BDI-II) by Types of Violence Exposure**									
**Violence Type**	**Group**	***n***	***Mean***	***SD***	***t***	***df***	***p***	**Mean Difference**	**95% CI**
Threat of violence	No	46	16.39	11.9	−1.546	149	0.124	−3.31	[−7.55, 0.92]
	Yes	105	19.7	12.21					
Physical violence	No	58	15.17	11.25	−2.876	149	0.005	−5.72	[−9.65, −1.79]
	Yes	93	20.89	12.27					
Sexual violence	No	46	11.89	9.71	−4.879	149	<0.001	−9.78	[−13.75, −5.82]
	Yes	105	21.68	11.98					
Anticipated violence	No	58	14.48	12.28	−3.48	149	<0.001	−6.84	[−10.72, −2.96]
	Yes	93	21.32	11.41					
**Differences in PTSD Symptoms (PCL-5) by Types of Violence Exposure**									
**Violence Type**	**Group**	***n***	***Mean***	***SD***	***t***	***df***	***p***	**Mean Difference**	**95% CI**
Threat of violence	No	46	21.15	18.06	−3.275	149	0.001	−11.07	[−17.75, −4.39]
	Yes	105	32.22	19.55					
Physical violence	No	58	22.03	17.04	−3.474	149	<0.001	−11.06	[−17.36, −4.77]
	Yes	93	33.1	20.17					
Sexual violence	No	46	16.43	14.44	−5.616	149	<0.001	−17.85	[−24.13, −11.57]
	Yes	105	34.29	19.31					
Anticipated violence	No	58	20.76	19.63	−4.194	149	<0.001	−13.13	[−19.32, −6.95]
	Yes	93	33.89	18.13					

Note. BDI-II = Beck Depression Inventory–II. PCL-5 = PTSD Checklist for DSM-5. Negative t-values indicate the group exposed to violence (Yes) had a higher mean score than the unexposed group (No).

**Table 4:** Hierarchical Multiple Regression Predicting PTSD Symptoms (N = 150).

**Model Set 1.1. Experienced Threats of Physical or Sexual Violence**					
**Predictor**	***B***	***SE B***	***β***	***p***	***95% CI***
**Block 1**					
Constant	20.87	2.55	-	<0.001	[15.83, 25.92]
Threatened violence	**7.13**	**1.83**	**0.305**	**<** **0** **.001**	**[3.52, 10.74]**
Model 1: R^2^ = 0.093, Adj. R^2^ = 0.087, F(1,148) = 15.23, *p* < 0.001					
**Block 2**					
Constant	22.61	6.62	-	<0.001	[9.51, 35.70]
Threatened violence	**6.85**	**1.87**	**0.294**	**<** **0** **.001**	**[3.16, 10.55]**
Age	−0.10	0.15	−0.053	0.509	[−0.38, 0.19]
Employment	−0.13	3.14	−0.003	0.966	[−6.35, 6.08]
Region: Northeast	−1.33	4.23	−0.027	0.754	[−9.69, 7.04]
Region: Midwest	4.21	4.29	0.082	0.329	[−4.28, 12.70]
Region: West	**9.87**	**4.47**	**0.183**	**0.029**	**[1.04, 18.70]**
Model 2: R^2^ = 0.133, Adj. R^2^ = 0.096, ΔR^2^ = 0.039, F(6,143) = 3.65, *p* = 0.002					
**Model Set 1.2. Experience Intersectional Physical Violence**					
**Predictor**	***B***	***SE B***	***β***	***p***	***95% CI***
**Block 1**					
Constant	22.99	2.36	-	<0.001	[18.34, 27.65]
Physical violence	**5.97**	**1.81**	**0.262**	**0.001**	**[2.39, 9.55]**
Model 1: R^2^ = 0.068, Adj. R^2^ = 0.062, F(1,148) = 10.87, *p* = 0.001					
**Block 2**					
Constant	28.21	6.33	-	<0.001	[15.71, 40.72]
Physical violence	**5.63**	**1.85**	**0.247**	**0.003**	**[1.98, 9.29]**
Age	−0.20	0.15	−0.111	0.172	[−0.49, 0.09]
Employment	0.75	3.18	0.019	0.814	[−5.53, 7.03]
Region: Northeast	−1.15	4.3	−0.023	0.789	[−9.64, 7.34]
Region: Midwest	4.6	4.35	0.09	0.292	[−4.00, 13.19]
Region: West	8.17	4.61	0.152	0.078	[−0.93, 17.28]
Model 2: R^2^ = 0.109, Adj. R^2^ = 0.071, ΔR^2^ = 0.040, F(6,143) = 2.91, *p* = 0.010					
**Model Set 1.3. Experience Intersectional Sexual Violence**					
**Predictor**	***B***	***SE B***	***β***	***p***	***95% CI***
**Block 1**					
Constant	17.8	2.41	-	<0.001	[13.03, 22.56]
Sexual violence	**10.13**	**1.76**	**0.428**	**<** **0** **.001**	**[6.65, 13.61]**
*Model 1: R^2^ = 0.183, Adj. R^2^ = 0.177, F(1,148) = 33.12, p < 0.001*					
**Block 2**					
Constant	16.28	6.35	-	0.011	[3.72, 28.83]
Sexual violence	**10.4**	**1.78**	**0.439**	**<** **0** **.001**	**[6.88, 13.92]**
Age	−0.04	0.14	−0.022	0.774	[−0.31, 0.23]
Employment	1.1	2.95	0.028	0.709	[−4.72, 6.93]
Region: Northeast	−2.04	3.97	−0.041	0.608	[−9.89, 5.80]
Region: Midwest	3.98	4.03	0.078	0.325	[−3.99, 11.95]
Region: West	**11.12**	**4.19**	**0.207**	**0.009**	**[2.84, 19.40]**
Model 2: R^2^ = 0.233, Adj. R^2^ = 0.201, ΔR^2^ = 0.051, F(6,143) = 7.26, *p* < 0.001					
**Model Set 1.4. Anticipated Intersectional Violence**					
**Predictor**	***B***	***SE B***	***β***	***p***	***95% CI***
**Block 1**					
Constant	13.72	3.26	-	<0.001	[7.28, 20.16]
**Anticipated violence**	**5.72**	**1.1**	**0.394**	**<** **0** **.001**	**[3.55, 7.88]**
Model 1: R^2^ = 0.155, Adj. R^2^ = 0.149, F(1,148) = 27.13, *p* < 0.001					
**Block 2**					
Constant	12.55	7.12	-	0.080	[3.72, 28.83]
**Anticipated violence**	**5.48**	**1.12**	**0.377**	**<** **0** **.001**	**[3.26, 7.70]**
Age	−0.05	0.14	−0.026	0.74	[−0.33, 0.23]
Employment	2.19	3.05	0.055	0.475	[−3.84, 8.21]
Region: Northeast	0.79	4.06	0.016	0.846	[−7.23, 8.81]
Region: Midwest	4.66	4.15	0.091	0.263	[−3.53, 12.86]
Region: West	8.75	4.34	0.163	0.045	[0.18, 17.33]
Model 2: R^2^ = 0.187, Adj. R^2^ = 0.153, ΔR^2^ = 0.032, F(6,143) = 5.48, *p* < 0.001					
**Model Set 1.5. Anticipated and Experienced Intersectional Violence**					
**Predictor**	***B***	***SE B***	***β***	***p***	***95% CI***
**Block 1—Violence Exposures Only**					
Constant	9.979	3.07	—	0.003	[3.53, 16.43]
Threat of violence	−0.391	4.2	−0.06	0.872	[−5.18, 4.40]
Physical violence	0.225	3.6	0.08	0.915	[–3.95, 4.40]
**Sexual violence**	7.872	**3.95**	**0.36**	<0.001	**[3.41, 12.34]**
**Anticipated violence**	3.973	**3.11**	**0.23**	<0.001	**[1.67, 6.28]**
Model 1 Fit: R^2^ = 0.232, Adjusted R^2^ = 0.211, F(4,145) = 10.93, *p* < 0.001					
**Predictor**	***B***	***SE B***	***β***	***p***	***95% CI***
**Block 2**					
Constant	6.227	6.991	—	0.375	[–7.59, 20.05]
Threat of violence	−0.404	2.450	−0.017	0.869	[−5.25, 4.44]
Physical violence	−0.745	2.203	−0.033	0.736	[–5.10, 3.61]
Sexual violence	**8.805**	**2.278**	**0** **.372**	**<** **0** **.001**	**[4.30, 13.31]**
Anticipated violence	**3.833**	**1.180**	**0** **.264**	**0** **.001**	**[1.50, 6.17]**
Age	0.027	0.138	0.015	0.843	[–0.25, 0.30]
Employment	2.112	2.912	0.054	0.470	[−3.65, 7.87]
Region: Northeast	−1.237	3.901	−0.025	0.752	[–8.95, 6.48]
Region: Midwest	3.792	3.933	0.074	0.337	[–3.99, 11.57]
Region: West	10.005	4.201	0.186	0.019	[1.70, 18.31]
Model 2 Fit: R^2^ = 0.261, Adjusted R^2^ = 0.213, ΔR^2^ = 0.029, F(9,140) = 5.48, *p* < 0.001					

**Table 5:** Hierarchical Multiple Regression Predicting Depressive Symptoms (N = 150).

**Model Set 2.1. Experienced Threats of Physical or Sexual Violence**					
**Predictor**	***B***	***SE B***	***β***	***p***	***95% CI***
**Model 1**					
Constant	16.03	1.63	—	<0.001	[12.81, 19.26]
Threat of violence	**2.36**	**1.17**	**0.164**	**0.045**	**[0.05, 4.67]**
**Model 1: R = 0.164, R^2^ = 0.027, Adj. R^2^ = 0.020, F(1,148) = 4.09, *p* = 0.045**					
**Model 2**					
Constant	17.1	4.3	—	<0.001	[8.61, 25.59]
Threat of violence	2.09	1.21	0.145	0.088	[−0.31, 4.48]
Age	−0.06	0.09	−0.052	0.543	[−0.24, 0.13]
Employment	0.17	2.04	0.007	0.932	[−3.86, 4.20]
Northeast	1.62	2.74	0.052	0.557	[−3.81, 7.04]
Midwest	1.62	2.79	0.051	0.563	[−3.89, 7.12]
West	3.81	2.9	0.115	0.19	[−1.91, 9.54]
Model 2: R = 0.204, R^2^ = 0.042, Adj. R^2^ = 0.001, F(6,143) = 1.03, *p* = 0.407					
**Model Set 2.2. Experience Intersectional Physical Violence**					
**Predictor**	***B***	***SE B***	***β***	***p***	***95% CI***
**Model 1**					
Constant	16.26	1.48	—	<0.001	[13.33, 19.19]
Physical violence	**2.46**	**1.14**	**0.175**	**0.032**	**[0.21, 4.72]**
**Model 1: R = 0.175, R^2^ = 0.031, Adj. R^2^ = 0.024, F(1,148) = 4.67, *p* = 0.032**					
**Model 2**					
Constant	18.46	4.04	—	<0.001	[10.47, 26.44]
Physical violence	2.27	1.18	0.161	0.056	[−0.06, 4.61]
Age	−0.09	0.09	−0.083	0.326	[−0.28, 0.09]
Employment	0.45	2.03	0.018	0.827	[−3.56, 4.45]
Northeast	1.47	2.74	0.048	0.592	[−3.95, 6.89]
Midwest	1.64	2.78	0.052	0.556	[−3.85, 7.12]
West	3.03	2.94	0.091	0.305	[−2.79, 8.84]
Model 2: R = 0.215, R^2^ = 0.046, Adj. R^2^ = 0.006, F(6,143) = 1.16, *p* = 0.332					
**Model Set 2.3. Experience Intersectional Sexual Violence**					
**Predictor**	***B***	***SE B***	***β***	***p***	***95% CI***
**Model 1**					
Constant	13.14	1.54	—	<0.001	[10.09, 16.19]
Sexual violence	**5.08**	**1.13**	**0** **.347**	**<** **0** **.001**	**[2.85, 7.30]**
**Model 1: R = 0.347, R^2^ = 0.121, Adj. R^2^ = 0.115, F(1,148) = 20.3, *p* < 0.001**					
**Model 2**					
Constant	12.32	4.16	—	0.004	[4.10, 20.54]
Sexual violence	**5.08**	**1.17**	**0** **.348**	**<0.001**	**[2.77, 7.39]**
Age	−0.02	0.09	−0.014	0.863	[−0.19, 0.16]
Employment	0.62	1.93	0.025	0.749	[−3.20, 4.44]
Northeast	0.87	2.60	0.028	0.739	[−4.27, 6.01]
Midwest	1.25	2.64	0.039	0.637	[−3.97, 6.47]
West	4.23	2.74	0.128	0.125	[−1.19, 9.66]
**Model 2: R = 0.369, R^2^= 0.136, Adj. R^2^= 0.10, F(6,143) = 3.8, *p* = 0.002**					
**Model Set 2.4. Anticipated Intersectional Violence**					
**Predictor**	***B***	***SE B***	***β***	***p***	***95% CI***
**Model 1**					
Constant	10.69	2.06	—	<0.001	[6.62, 14.76]
Anticipated violence	**3.02**	**0.69**	**0.337**	**<** **0** **.001**	**[1.65, 4.39]**
**Model 1: R = 0.337, R^2^ = 0.113, Adj. R^2^ = 0.107, F(1,148) = 18.94, *p* < 0.001**					
**Model 2**					
Constant	9.52	4.56	—	0.038	[0.51, 18.53]
Anticipated violence	**2.96**	**0.72**	**0.33**	**<** **0** **.001**	**[1.54, 4.38]**
Age	−0.01	0.09	−0.011	0.888	[−0.19, 0.17]
Employment	1.22	1.95	0.05	0.532	[−2.64, 5.08]
Northeast	2.25	2.6	0.073	0.387	[−2.88, 7.38]
Midwest	1.54	2.65	0.049	0.563	[−3.71, 6.78]
West	2.97	2.78	0.089	0.287	[−2.52, 8.45]
**Model 2: R = 0.354, R^2^ = 0.126, Adj. R^2^ = 0.089, F(6,143) = 3.42, *p* = 0.003**					
**Model Set 2.5. Anticipated and Experienced Intersectional Violence**					
**Predictor**	***B***	***SE B***	***β***	***p***	***95% CI***
**Block 1**					
Constant	9.297	2.094	—	<0.001	[5.16, 13.44]
Threat of violence	−2.364	1.554	−0.164	0.131	[−5.44, 0.71]
Physical violence	0.064	1.354	0.005	0.962	[−2.61, 2.74]
Sexual violence	**5.098**	**1.449**	**0** **.349**	**<0.001**	**[2.23, 7.96]**
Anticipated violence	**2.421**	**0** **.749**	**0** **.270**	**0** **.002**	**[0.94, 3.90]**
**Model 1: R^2^ = 0.204, Adjusted R^2^ = 0.182, F(4,145) = 9.30, *p* < 0.001**					
**Predictor**	***B***	***SE B***	***β***	***p***	***95% CI***
**Block 2**					
Constant	6.800	4.567	—	0.139	[−2.23, 15.83]
Threat of violence	−2.500	1.601	−0.174	0.121	[−5.66, 0.67]
Physical violence	−0.344	1.439	−0.024	0.812	[−3.19, 2.50]
Sexual violence	**5.395**	**1.488**	**0** **.369**	**<0.001**	**[2.45, 8.34]**
Anticipated violence	**2.456**	**0** **.771**	**0** **.274**	**0** **.002**	**[0.93, 3.98]**
Age	0.019	0.090	0.017	0.837	[−0.16, 0.20]
Employment	1.596	1.902	0.066	0.403	[−2.17, 5.36]
Region: Northeast	1.673	2.548	0.054	0.512	[−3.36, 6.71]
Region: Midwest	1.350	2.570	0.043	0.600	[−3.73, 6.43]
Region: West	3.821	2.745	0.115	0.166	[−1.61, 9.25]
**Model 2: R^2^ = 0.212, Adjusted R^2^ = 0.161, ΔR^2^ = 0.008, F(9,140) = 4.18, *p* < 0.001** Note. Significant models and variables are bolded.					

## Data Availability

The data presented in this study are available on request from the corresponding author due to privacy restrictions.

## Abbreviations

The following abbreviations are used in this manuscript:

BDI-II	Beck Depression Inventory II
PCL-5	Post-Traumatic Stress Symptom Checklist
PTSD	Post-traumatic stress disorder
SD	Standard deviation
SGM	Sexual and gender minorities
TGD	Transgender and gender diverse
US	United States
USTS	United States Transgender Survey
